# Building reproducible bridges to cross the “valley of death”

**DOI:** 10.1172/JCI177383

**Published:** 2024-01-02

**Authors:** Timothy M. Errington

**Affiliations:** Center for Open Science, Charlottesville, Virginia, USA.

Research is cumulative. New investigations build on, challenge, or qualify claims based on prior evidence. When we have limited evidence, it is normal that some explanations are wrong and need to be refined or reconsidered. This self-corrective process is a hallmark of research. A key part of a healthy self-corrective process is that the evidence used for generating and refining explanations is credible and trustworthy. A weak foundation of evidence allows explanations to be generated that describe phenomena that are not occurring as well as inaccurate explanations of phenomena that do occur. We have made much progress in evaluating the credibility of the evidence foundation in research, and these investigations suggest that there is substantial room to improve the credibility and trustworthiness of research (1). If we can improve the foundation of evidence upon which explanations are built and debated, then we might dramatically accelerate the pace of discovery of knowledge, treatments, and solutions in service of humanity.

## Replicability challenges in the drug-development pipeline

When considering the drug-development pipeline, the overall failure rate of drugs that passed into phase 1 trials to final approval is 90% (2). This number does not include the failure rate in the preclinical stage, which is unknown. This lack of translation from promising preclinical findings to success in human trials is known as the “valley of death” (3). What might be some of the reasons for this failure rate? One reason is that translation of models to humans is challenging and we are limited by our scientific knowledge and methodological approaches. Another reason is friction in the goals and incentives of preclinical research compared with the clinical phase and that we are pushing drugs into human trials when there is not enough confidence in the support for them.

As an example of this friction, in 2014, the ALS Therapy Development Institute performed replications of more than 100 promising drug candidates in an established mouse model of ALS (4). In all cases, the replications fell well short of the exciting published findings. These potential therapies also had disappointing results in human testing. This poor correlation between preclinical findings and clinical outcomes has also been observed in other disease areas, such as glioblastoma (5). Poor replicability leads to wasting time, money, and animals trying to build on evidence that was not as reliable or credible as it appeared in the original research. Furthermore, false hope can even lead to clinical trials that would not have been pursued if the lack of replicability had been known in advance. Similar replicability challenges were observed in the Reproducibility Project: Cancer Biology, which performed replications of 50 key preclinical cancer biology experiments (6). On average, replication effect sizes were 85% smaller than original effect sizes. As an example, an original finding reported a potential therapeutic effect that decreased the average tumor growth in a mouse model by 57% compared with control, whereas the replication of that finding found it to be 7%. Even more worrisome is that the majority of papers lacked transparency of the underlying data, analysis, materials, and methods of the experiments (7). This lack of transparency has large implications for the confidence one has as these potential therapies move through the drug-development pipeline. While there have been improvements since these studies to increase rigor and reproducibility standards, such as many journals increasing reporting requirements regarding methods, there is still an implementation hurdle, with many preclinical studies lacking sufficient detail (8).

Driving factors for a lower than desired level of replicability include shortcomings in the design, conduct, and communication of research, such as small sample sizes, lack of blinding and randomization, reagent quality control issues, and transparency of methods and results (9). Whether these issues arise from lack of knowledge, inadequate funding, perverse incentives, sloppiness, or biases, it results in an ever-growing body of evidence that makes it challenging to distinguish between those findings that will replicate and translate and those that will not. Thus, a challenge when trying to cross the “valley of death” is how to distinguish between what preclinical research warrants the investment to continue advancing to human testing and what research should be stopped.

What are our options? We could accept the high failure rate of the drug-development pipeline as it operates now and continue to invest more in producing research with known low rates of replicability and translatability in hopes that we’ll increase the number of viable treatments that successfully reach the public. If we continue with this path, it means spending more money and time and asking more patients to take on the risk of enrolling in trials where we know there will be a higher than desired rate of failure. Alternatively, we can change where failure occurs in the pipeline, aiming to detect failure at the preclinical step and thus decreasing the failure rate for drugs that reach human trials. This shift means testing and implementing innovative processes that can increase confidence in which promising leads warrant further investment. It means integrating more clinical insights earlier into the preclinical pipeline through directed collaborative efforts that can better inform how to design, measure, and model human disease. By closing this translational gap from both ends, we create a bridge instead of tossing preclinical findings over a chasm hoping they survive if they make it to the other side.

## What does a reproducible bridge look like?

Enhancing the replicability of preclinical research starts by improving the methods of conducting research and sharing findings. Such efforts include, but are not limited to, increasing sample sizes; better descriptions and sharing of data, code, protocols, and materials; and incorporating bias-reducing mechanisms such as blinding, randomization, and preregistration (1). These changes are vital to increasing the credibility of preclinical research, yet wide-scale implementation is incredibly challenging because it requires coordination of many entities in a decentralized system (e.g., funders, institutions, researchers, publishers). Improvements to help increase the methods and transparency of research can largely leverage existing training and tools, though support to maintain, improve, and expand them is needed. Support for these initiatives and enabling their integration within existing structures (e.g., graduate curriculum) would have a substantial impact beyond just investing in the research itself. While there is still a need for this type of investment, there are examples of these changes taking place, such as through the NIH Generalist Repository Ecosystem Initiative (10) and the Chan Zuckerberg Initiative’s (CZI’s) Essential Open Source Software For Science program (11). Furthermore, improving methods and transparency is not enough. No single finding provides definitive evidence — inherently we need multiple lines of evidence (12). Simultaneously, we need to continuously confront how replicable, and thus reliable, each of these lines of evidence is (13). How might that look in practice?

One way is to stop treating preclinical research as a single phase in the drug-development pipeline. Instead, we need to incorporate structured phases to help assess whether to proceed forward or not, similarly to how we have multiple phases of clinical research. For example, requiring successful replication of key findings by independent laboratories before the drug candidate proceeds would increase confidence in the findings (14, 15). As an example of how this can look in practice, additional funding would be contingent on replication success, such as with the NIH Somatic Cell Genome Editing (SCGE) Consortium (16). This requirement could then be expanded to require successful replication by multiple independent laboratories (17). Finally, observing similar results when testing findings in other valid preclinical models would increase confidence that the findings are generalizable and uncover what limitations there might be (18). The Stroke Pre-Clinical Assessment Network (SPAN) is an example of how this can look in practice, having recently completed an investigation of six candidate therapies that underwent replication in multiple laboratories using different rodent models of ischemic stroke (19). Overall, the aim of an approach like this would be to strengthen translatability by continuing to increase heterogeneity and confidence in replicability throughout the preclinical process. It would also create a stronger mechanism for identifying which findings to pursue and which not to pursue.

The bridge from preclinical to clinical can also be built from the clinical side of the drug-development pipeline. Clinical insights need to inform preclinical designs, and preclinical models and measurements need to be similar to what is observed in humans. Similarly, evaluation processes for assessing the clinical promise of potential therapies using preclinical evidence could benefit from consideration of both preclinical and clinical insights. This evaluation could include whether preclinical models were successfully replicated in more than one model and/or by more than one laboratory and how similar agents fared in clinical development (20).

## Conclusions

While adding more requirements to the drug-development pipeline might seem counterintuitive to progress, such changes are necessary to better discern what areas are suitable for further advancement. If more effort is invested into optimizing the earlier stages of the drug development pipeline rather than rushing prematurely to human trials, the failure rate of drug candidates at the clinical stages would decrease. Some failing is necessary to finding what’s right, but we need to create a system that can robustly substantiate novel claims and tolerates observing failure earlier in the pipeline.

## Version 1 01/02/2024

Electronic publication
